# Combination of Machine Learning and Raman Spectroscopy for Determination of the Complex of Whey Protein Isolate with Hyaluronic Acid

**DOI:** 10.3390/polym16050666

**Published:** 2024-02-29

**Authors:** Oksana A. Mayorova, Mariia S. Saveleva, Daniil N. Bratashov, Ekaterina S. Prikhozhdenko

**Affiliations:** Science Medical Center, Saratov State University, 83 Astrakhanskaya Str., 410012 Saratov, Russia

**Keywords:** Raman spectroscopy, whey protein isolate, hyaluronic acid, random forest, gradient boosting, principal component analysis, feature importance

## Abstract

Macromolecules and their complexes remain interesting topics in various fields, such as targeted drug delivery and tissue regeneration. The complex chemical structure of such substances can be studied with a combination of Raman spectroscopy and machine learning. The complex of whey protein isolate (WPI) and hyaluronic acid (HA) is beneficial in terms of drug delivery. It provides HA properties with the stability obtained from WPI. However, differences between WPI-HA and WPI solutions can be difficult to detect by Raman spectroscopy. Especially when the low HA (0.1, 0.25, 0.5% w/v) and the constant WPI (5% w/v) concentrations are used. Before applying the machine learning techniques, all the collected data were divided into training and test sets in a ratio of 3:1. The performances of two ensemble methods, random forest (RF) and gradient boosting (GB), were evaluated on the Raman data, depending on the type of problem (regression or classification). The impact of noise reduction using principal component analysis (PCA) on the performance of the two machine learning methods was assessed. This procedure allowed us to reduce the number of features while retaining 95% of the explained variance in the data. Another application of these machine learning methods was to identify the WPI Raman bands that changed the most with the addition of HA. Both the RF and GB could provide feature importance data that could be plotted in conjunction with the actual Raman spectra of the samples. The results show that the addition of HA to WPI led to changes mainly around 1003 cm^−1^ (correspond to ring breath of phenylalanine) and 1400 cm^−1^, as demonstrated by the regression and classification models. For selected Raman bands, where the feature importance was greater than 1%, a direct evaluation of the effect of the amount of HA on the Raman intensities was performed but was found not to be informative. Thus, applying the RF or GB estimators to the Raman data with feature importance evaluation could detect and highlight small differences in the spectra of substances that arose from changes in the chemical structure; using PCA to filter out noise in the Raman data could improve the performance of both the RF and GB. The demonstrated results will make it possible to analyze changes in chemical bonds during various processes, for example, conjugation, to study complex mixtures of substances, even with small additions of the components of interest.

## 1. Introduction

In the study of macromolecule interaction, the formation of complexes and conjugates is an important task in various fields, such as targeted drug delivery [1,2,3], tissue regeneration [4,5], and the food industry [6,7]. One of the approaches to studying the chemical composition of substances is Raman spectroscopy [8,9,10]. This method can be implemented to collect data on materials in various aggregation states with little or no need for sample preparation. However, to process the Raman data of macromolecules and their mixtures or complexes, machine learning approaches should be used since there will be small differences in their Raman spectra [11].

Classification or regression models can be applied depending on the task at hand, i.e., whether it is necessary to distinguish between several classes or to study the changes that occur when different amounts of a substance of interest are added to the matrix. Models based on the support vector machine method are usually applied in this case [12,13]. The main disadvantage is that although it is possible to build a model with high accuracy (>90%), the result will be data clouds that do not explain the chemical bonds that most affect the classification/regression process. Another approach is principal component analysis (PCA), which provides the Raman spectra of its principal components with decreasing variance explained in the data [14,15]. However, the spectra of the components are synthetic and mathematically calculated and have Raman bands with negative intensity, which can lead to confusion in the case of determining chemical bonds. The multidimensional curve resolution approach overcomes this disadvantage by implementing non-negative constraints on both the spectral and concentration profiles [16]. PCA and multidimensional curve resolution are excellent for analyzing Raman spectra of mixtures or complex systems, such as cells [16,17], since there are different substances in comparable amounts. In this case, the calculated Raman spectra of the components can also be spatially separated. However, when comparing the Raman spectra of a macromolecule and its complex, these approaches cannot single out the Raman band that changes the most due to chemical bonding. Nevertheless, such dimensionality reduction approaches can be implemented as an intermediate step to reduce noise in the spectra while maintaining a large amount of explained variance in the data [18]. In the simplest way to obtain the chemical bond of the macromolecule most altered by complexation, the difference spectrum between the average normalized spectra of the classes of interest can be calculated [19]. Thus, the Raman spectra range with the greatest changes can be determined to further approximate each Raman band in terms of its width, intensity, and peak position [20]. The main disadvantage of this approach is that it involves the least amount of automation compared with other considered methods.

Hyaluronic acid (HA) is a natural polyanion consisting of disaccharide units of D-glucuronic acid and D-N-acetylglucosamine connected by the glycosidic bonds beta-1,4 and beta-1,3 [21]. HA is an important component of the mucin layer of mucous membranes that are present in the connective tissues of animals and humans [22]. In medicine, low-molecular-weight HA is widely used in tissue regeneration, the removal of free radicals, and anti-inflammatory and antitumor therapy. However, its rapid in vivo degradation [23,24], low stability, and inability to form aggregates in aquatic solutions complicate its use [23,24,25,26]. The functional modification of HA by interacting with proteins opens up great opportunities for its use as a therapeutic agent [27]. The formation of intermolecular hydrogen bonds, as well as various complexes due to electrostatic and van der Waals interactions between proteins and HA, opens up new possibilities for use in medicine. Whey protein isolate (WPI) is a cheap precursor used to develop targeted delivery systems and a by-product of cheesemaking [28,29]. WPI is most often used as an emulsifier, gelling agent, and water-binding agent in the food, pharmaceutical, and cosmetic industries [30,31,32,33]. The main advantage of using WPI is its nontoxicity and biocompatibility.

Adding a small amount of HA to WPI can give the advantages of HA to the resulting mixture without a significant increase in price, which can be crucial in the production of targeted drug delivery systems. The use of a WPI-HA complex instead of WPI as a stabilizing agent can provide greater durability to the produced microcarriers [34]. Combinations of WPI and HA in various ratios and the properties of the resulting microgels and nanoparticles were previously described by Weigang Zhong et al., with the lowest amount of HA discussed at a protein:polysaccharide ratio of 10:1 [35,36,37]. Recent studies examined in detail the mechanism of interaction between WPI and HA. According to Fourier-transform infrared spectroscopy data, molecules form a complex with each other due to non-covalent interactions, namely, the formation of hydrogen bonds and electrostatic and hydrophobic interactions. In this case, no new covalent bonds are observed [34].

In this study, Raman spectroscopy was used in the analysis of WPI-HA conjugates with protein:polysaccharide ratios of 10:1, 20:1, and 50:1. Two ensemble methods based on decision trees, namely, random forest (RF) [38,39] and gradient boosting (GB) [40], were implemented to process the data obtained. Feature importance calculated from the regression and classification models was used to determine the Raman bands that changed the most between samples. The impact of noise reduction using PCA on the performance of both machine learning methods was assessed. The ability of the models to distinguish between WPI and WPI-HA conjugates was tested.

## 2. Materials and Methods

### 2.1. Materials

The whey protein isolate (WPI) was manufactured by California Gold Nutrition^®^ (Irvine, CA, USA) and purchased online from iHerb. Hyaluronic acid sodium salt (HA, purity of 99%, 404 $M_{W}$) was purchased from Macklin Biochemical Co., Ltd. (Shanghai, China).

Millipore Milli-Q water (18.2 MΩ·cm^−1^) was used as an aqueous medium during all sets of experiments.

### 2.2. Preparation of WPI-HA Conjugates

The concentration of WPI used to obtain the WPI-HA conjugate was the same in all cases (10% *w*/*v*). The WPI-HA conjugates were prepared in the course of mixing the WPI and HA solutions in saline (0.15 M NaCl). To prepare the conjugates with different HA concentrations, equal volumes of HA solution with specific HA concentrations were added to the WPI solutions. After that, the mixture was vigorously shaken for 30 min at 22 °C. The resulting WPI-HA complexes contained different concentrations of HA: 0.1, 0.25, and 0.5% *w*/*v*. The WPI-HA conjugates obtained were washed to remove the free HA by dialysis against saline for 3 days at 4 °C. A control sample of WPI was prepared by diluting the initial WPI solution twice with saline. Thus, 4 samples were prepared: WPI, WPI + 0.1% HA, WPI + 0.25% HA, and WPI + 0.5% HA.

### 2.3. Raman Scattering Measurements

A total of 10 μL of WPI-HA conjugates in various ratios were placed on a quartz substrate and dried in air before the Raman measurements were taken. After drying, the WPI solution and WPI-HA complexes formed thin films without the coffee ring effect. A Renishaw inVia confocal spectrometer (Renishaw, Wotton-under-Edge, UK) equipped with 532 nm laser was used to collect the Raman spectra of the dried WPI/HA mixture. All measurements were done with a 50×/0.5 N.A. objective and 2.5 mW laser power. Raman maps (600 single spectra, 20 × 30 points with 2 μm step) were collected from each sample.

### 2.4. Data Analysis

Raman measurement data was collected with Renishaw WiRE v.4.2 software (Renishaw, UK). If necessary, the Cosmic Ray Removal tool from Renishaw WiRE was applied to the obtained spectra. The polynomial background was removed from the collected Raman maps with the Subtract Baseline tool using a ten-degree polynomial. Further data processing was carried out using Python 3.6 in the Jupyter Notebook environment.

RenishawWiRE v.0.1.16 was used to import the Raman data. Spectra were normalized by the $L_{2}$-norm using the normalize method of sklearn.preprocessing. The Raman intensities in the wavenumber range of 750–1800 cm^−1^ were used in the regression and classification with random forest and gradient boosting. The data were split into two sets using the sklearn.model_selection.train_test_split method: 75% or 25% of the data (450 or 150 out of 600 spectra for each amount of HA) was used to train or test the estimators, respectively.

Principal component analysis using sklearn.decomposition.PCA with n_components set to 0.95 (retained 95% of the explained variance in the data) was performed to filter the noise from the Raman spectra.

RandomForestRegressor, RandomForestClassifier, GradientBoostingRegressor, and GradientBoostingClassifier estimators of sklearn.ensemble with 100 decision trees each and the max_depth hyperparameter set to 3 were used to evaluate the data. Different metrics, such as r2_score, accuracy_score, and confusion_matrix, of sklearn.metrics were used to assess the performances of the models. For all the randomized modules, the random_state hyperparameter was set to 5.

## 3. Results and Discussion

WPI-HA conjugates with different amounts of HA and control WPI solution were prepared. The WPI concentration remained the same in all the samples (5% *w*/*v*). Three amounts of HA added were used to make conjugates: 0.1% (WPI + 0.1% HA, 5%:0.1%, 50:1 protein:polysaccharide ratio), 0.25% (WPI + 0.25% HA, 5%:0.25%, 20:1 protein:polysaccharide ratio), and 0.5% (WPI + 0.5% HA, 5%:0.5%, 10:1 protein:polysaccharide ratio). The Raman spectra were measured to determine the effect of a small addition of HA on a protein molecule.

Samples (10 μL each) were placed on the quartz substrate and air dried. After this, the sample droplets formed thin films on the substrate without causing the coffee ring effect. The Raman maps (20 × 30 grid, 2 μm step) were collected from each sample. Thus, the entire collected dataset consisted of 2400 Raman spectra (600 spectra per sample). The Raman maps of several bands are shown in Figure S1 (Supplementary Materials).

The following preprocessing procedures were carried out: polynomial baseline removal and normalization. The Raman spectra were then truncated to the range in which Raman signals were observed: 750–1800 cm^−1^, with 626 intensity values per spectrum. The normalized intensity values of each wavenumber were considered features.

Before the introduction of the machine learning approaches, the dataset was divided into training and test datasets in a ratio of 75%:25%. Model fitting was carried out on the training dataset and model performance was evaluated on the test dataset. Both the regression and classification models were made based on the following ensemble methods: random forest (RF) [41] and gradient boosting (GB) [42]. Both of these approaches are based on decision trees. However, the distinction lies in the manner in which the ultimate outcome is produced. In a random forest, the decision trees function simultaneously, and the final prediction is obtained by averaging. On the other hand, in gradient boosting, the decision trees function sequentially, improving the prediction and reducing the error at each iteration. The number of decision trees in RF and GB was set at 100 for the regression and classification problems.

**Figure 1:**
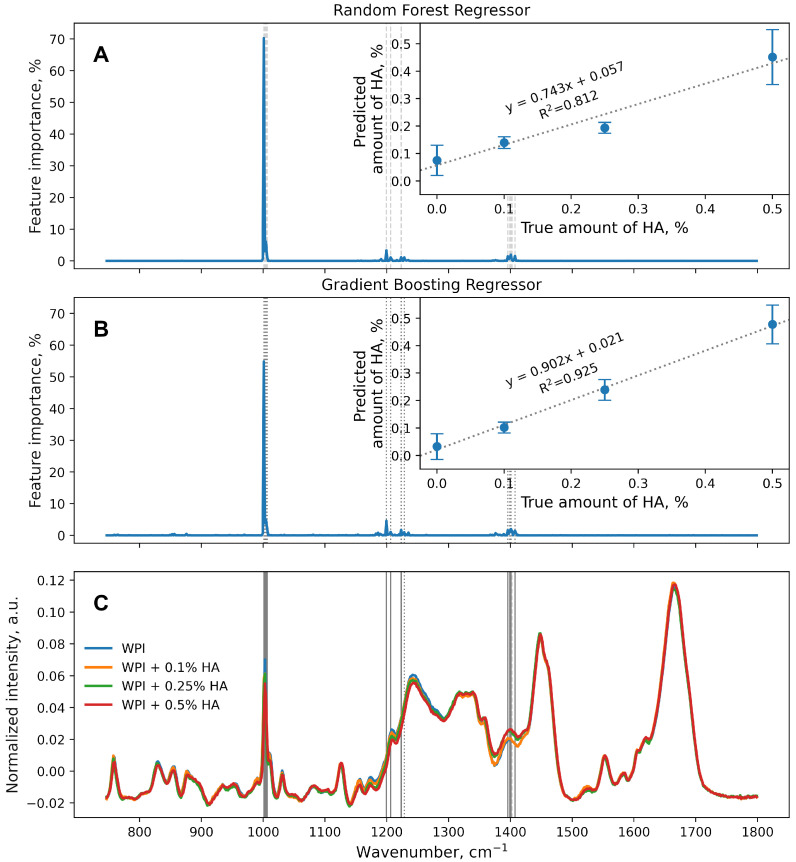
Feature importances calculated by RandomForestRegressor (**A**) and GradientBoostingRegressor (**B**) estimators, respectively. The insets in (**A**,**B**) correspond to predicted HA amount for test dataset (150 samples per true HA amount value) with the estimated linear regression curves. Markers and error bars correspond to the mean and standard deviation, respectively. (**C**) Average of 600 normalized spectra per true HA amount value. Grey dashed and dotted lines correspond to wavenumbers with feature importance greater than 1%. Matching lines in (**A**,**B**) are indicated as solid lines in (**C**).

The results of the regression models are presented in Figure 1. To evaluate the performance of both regression models, the determination coefficients (R^2^) were calculated for the resulting linear regression curves. Since both estimators were implemented with the same set of hyperparameters, the comparison of their performance was considered valid. In general, the RF (Figure 1A) was not as accurate as the GB (Figure 1B), especially for intermediate amounts of HA (0.1% and 0.25%). The HA amounts predicted by the RF were as follows: 0.075 ± 0.055% (WPI), 0.140 ± 0.022% (WPI + 0.1% HA), 0.194 ± 0.020% (WPI + 0.25% HA), and 0.452 ± 0.100% (WPI + 0.5% HA). Thus, averaging the predictions made by 100 individual decision trees, which was called an RF, was insufficient for the current dataset. On the other hand, the stage-wise optimization of 100 individual decision trees (GB) provided better results, with R^2^ = 0.925.

RandomForest and GradientBoosting estimators from sklearn.ensemble allowed is to obtain feature importance data, i.e., which feature (wavenumber of the spectra) influenced the data analysis results the most (Figure 1A,B). These data could be compared with the actual Raman spectra of the WPI-HA conjugates (Figure 1C).

One can see that not all of the Raman active bands of the spectra impacted the data analysis with either RandomForestRegressor or GradientBoostingRegressor. Thus, oscillations of the WPI molecule could be observed, which were primarily influenced by the addition of HA and subsequent conjugation. Features with an importance value higher than 1% are highlighted with grey lines in Figure 1. The averaged Raman spectra per sample, with the respective standard deviations, are shown in Figure S2 (Supplementary Materials). Although the performances of both types of estimators varied, most of the important features were the same, with a few exceptions: 1228 cm^−1^ (RF only) and 1403 cm^−1^ (GB only). However, these particular features were not located on the specific Raman peaks that may be associated with the chemical bonds of either WPI or HA. Additional peaks were also not found in any sample while analyzing the entire dataset (see Figure S2 in the Supplementary Materials). Thus, these particular features (1228 cm^−1^ and 1403 cm^−1^) could be considered as calculation artifacts. The wavenumbers of the Raman bands with the corresponding chemical bonds are given in Table 1.

The interactions between WPI and HA were recently studied with Fourier-transform infrared spectroscopy (FTIR) [34]. According to this research, the FTIR spectra of the WPI and WPI-HA complexes were similar; however, the slight shifts of amide I from 1651 cm^−1^ to 1644 cm^−1^ and amide II from 1536 cm^−1^ to 1543 cm^−1^ were observed with higher HA contents (2.5:1 ratio of WPI:HA). WPI and HA form noncovalent bonds and the increase in the amount of HA leads to the transformation of the α-helical structure of WPI to an amorphous β-sheet structure, followed by the formation of hydrogen bonds and strong hydrophobic interactions between WPI and HA [44].

However, no changes were observed in the Raman bands of amide I and amide II while solving the regression task, which could be explained by the lower amounts of HA in the WPI-HA complexes discussed. The most influential Raman band found using RandomForestRegressor or GradientBoostingRegressor was 1003 cm^−1^, which corresponds to phenyl ring angular bending vibrations. This band was not discussed by Wang et al. [34], as the assigned oscillation is symmetrical, and thus, is not FTIR active. Thus, it can be assumed that the addition of hyaluronic acid to the whey protein isolate, even in the smallest amount, affected this specific amino acid, which resulted in changes in its vibrations that could be detected using a combination of Raman spectroscopy and machine learning approaches.

**Figure 2:**
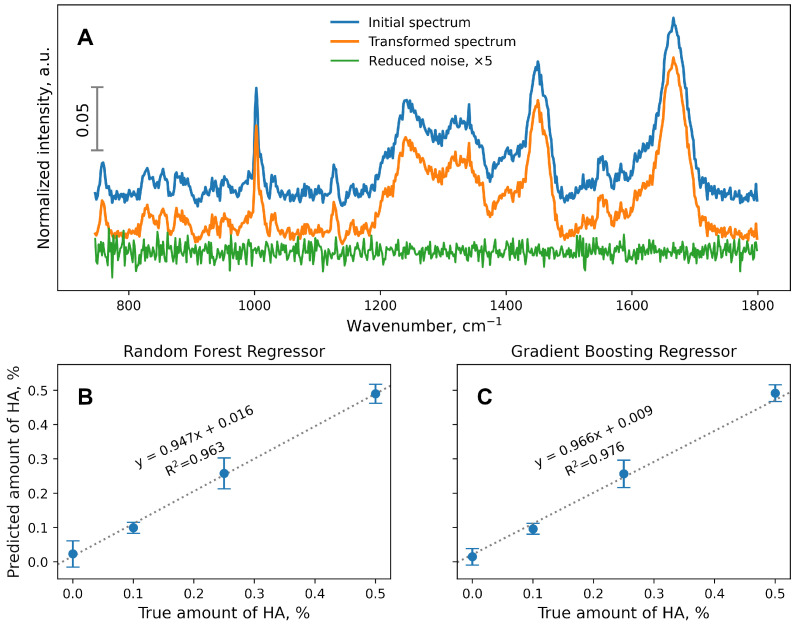
(**A**) Random spectrum of a dataset before PCA implementation (initial spectrum), inverted after PCA transformation (noise-reduced, transformed spectrum), and difference between original and transformed spectra (reduced noise). The intensity of the noise spectrum was multiplied by 5 for clarity. The vertical scale corresponds to the normalized intensity. Linear regression curves calculated from noise-reduced data with RF (**B**) and GB (**C**) estimators.

To improve the performance of estimators, dimensionality reduction procedures can be implemented, for instance, principal component analysis (PCA). PCA provides a projection of a data set from a multidimensional space (according to the number of features in the data set) to lower-dimensional space. Thus, each individual sample in a data set can be estimated as a linear composition of principal components with associated coefficients. The number of components must be less than or equal to the number of features in the original data set. In the sklearn.decomposition.PCA module, the number of components can also be set indirectly by using a fraction corresponding to the amount of variance to be explained. In the current data set, which originally contained 626 features, setting the PCA n_components to 0.95 resulted in the retention of 480 features in the transformed data set. The transformed spectra can be restored to the original number of features with the inverse_transform method of sklearn.decomposition.PCA. This approach can be used to obtain visual information on the amount of data remaining or lost during the PCA (Figure 2A).

The dataset with a reduced number of features was employed to train and evaluate the RF (Figure 2B) and GB (Figure 2C) regression estimators. Although the amount of noise was not significantly reduced compared with the original Raman spectra (Figure 2A), the performances of the RF- and GB-based regression models were significantly better than using the original dataset (Figure 1A,B). However, this approach had a serious drawback because, after denoising, the transformed features of the dataset did not correlate with the original features (wavenumbers). It was not feasible to determine the relationship between particular chemical bond vibrations and their influences on the RF or GB performances.

Examining the influence of the HA amount on the Raman spectra of WPI could be addressed not only through regression but also through classification. However, this approach restricted the capacity to utilize calibration curves to identify unknown amounts of HA that were not the same as those used in the estimation procedure. Each additional HA amount required another version of the classification estimator for training.

**Figure 3:**
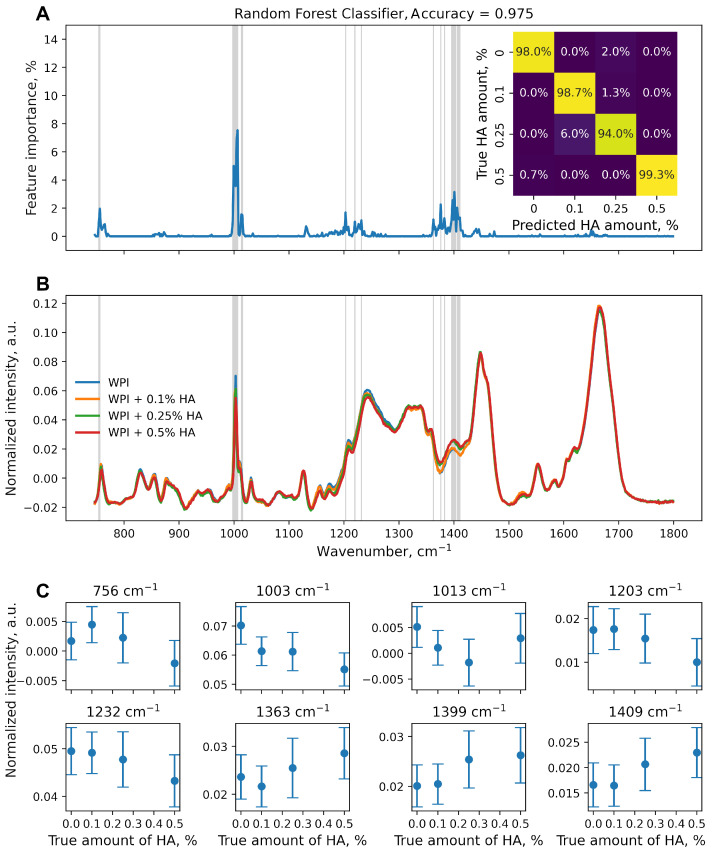
(**A**) Feature importance calculated from RandomForestClassifier estimator. Confusion matrix of the estimator for test data set normalized over the true values is shown in the inset. (**B**) Average of 600 normalized spectra per true HA amount value. Grey lines on (**A**,**B**) correspond to wavenumbers with feature importance greater than 1%. (**C**) Mean normalized intensities with standard deviations (600 spectra per true HA amount value) for Raman bands marked with grey in (**A**,**B**).

**Figure 4:**
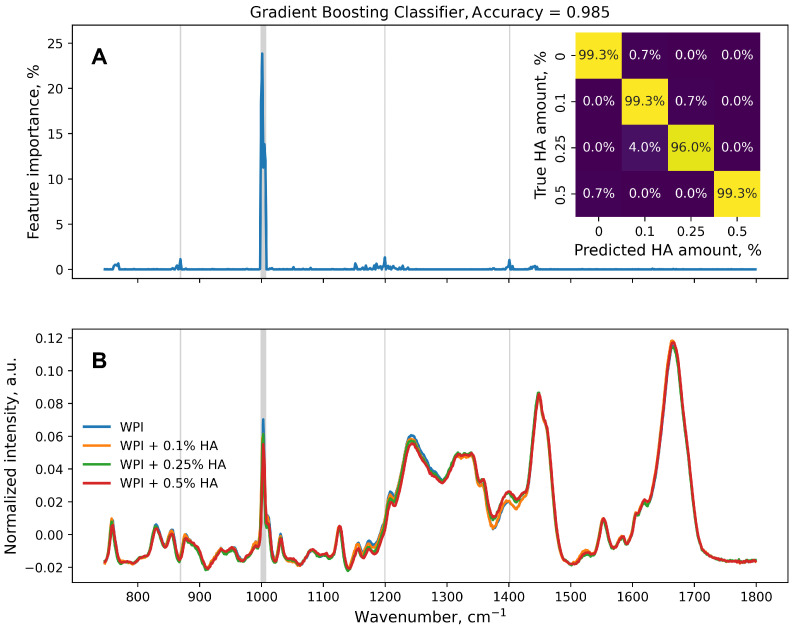
(**A**) Feature importance calculated from GradientBoostingClassifier estimator. Confusion matrix of the estimator for test data set normalized over the true values is in the inset. (**B**) Average of normalized 600 spectra per true HA amount value. Grey areas and lines in (**A**,**B**) correspond to wavenumbers with feature importance greater than 1%.

Nonetheless, two classification estimators based on the RF (Figure 3) and GB (Figure 4) were trained on the original dataset (626 features, 600 samples per class, train:test ratio of 75%:25%). The performance of the classification model was higher than that of the same regression model. Changing the task to classification made the trained models more sensitive, as more features of the original dataset were found to be important (Figure 3A and Figure 4A). Lines in gray in Figure 3A,B and Figure 4 correspond to wavenumbers at which the feature importance exceeded 1%.

However, the graph of the normalized intensity depending on the amount of HA at the selected wavenumbers may not be so informative (Figure 3C). Noticeable changes could be seen at 1003 cm^−1^, although there was no difference between the WPI + 0.1% HA and WPI + 0.25% HA samples. For 1203 and 1232 cm^−1^, there were no significant differences in the normalized intensity between three of the samples with the lowest-to-zero HA amounts. For 756, 1013, and 1363 cm^−1^, the change in the normalized intensity was non-linear. For 1399 and 1409 cm^−1^, there was a noticeable difference between the group of WPI, WPI + 0.1% HA, WPI + 0.25% HA, and WPI + 0.5% HA samples, with no differences between samples within the group.

Although no additional chemical bonds are formed in the WPI-HA complex, the hydrogen bonds between the HA and WPI molecules result in changes in the Raman bands of the WPI [45]. The intermolecular hydrogen bonds cause several molecules to associate, thus reducing the vibration frequencies of C=O stretching (∼1400 cm^−1^) and complex ring vibrations (e.g., ∼760 cm^−1^ and 1003 cm^−1^). However, these effects could not be visualized directly with the WPI-HA Raman band studies, as the amount of HA used was low.

Considering the performance of both classification models, the classes were clearly distinguishable; mislabeling of spectra mostly occurred for WPI + 0.25% HA samples in 6.0% and 4.0% of cases for the RF (Figure 3A, inset) and GB (Figure 4A, inset), respectively. The RandomForestClassifier was found to be the most sensitive model that was trained on the original data set, as more features were considered important for the analysis. The Raman band assigned to amide I had non-zero importance (Figure 3A), which was not observed for any other model trained in the present study. Thus, the amide C=O stretching also changed with the amount of HA, which correlates with Wang et al.’s FTIR spectra research [34].

Thus, models based on the random forest or gradient boosting and the feature_importances_ property made it possible to determine the area with the greatest changes in the WPI spectra with the addition of HA in different amounts, which were not noticeable in the direct assessment of Raman intensities.

**Figure 5:**
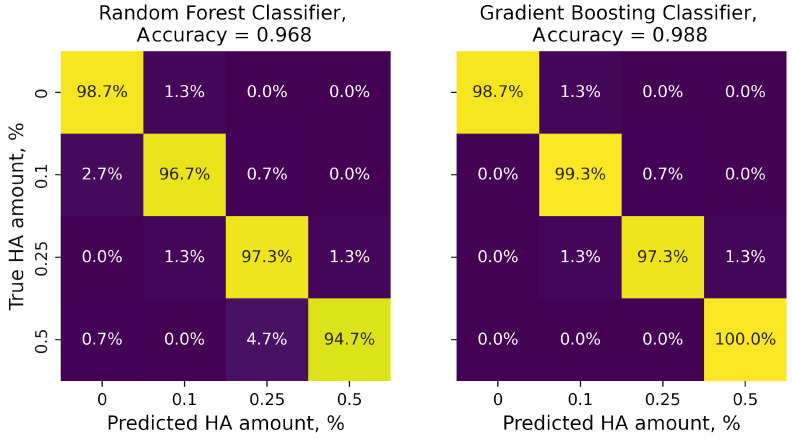
Confusion matrices of RF and GB estimators fitted using denoised-with-PCA Raman spectra. Values were normalized over the true HA amount.

The training of both RandomForestClassifier and GradienBoostingClassifier on the denoised-with-PCA data set resulted in a different outcome (Figure 5). The accuracy of the GB estimators was slightly improved from 0.985 to 0.988, while the accuracy of the RF models decreased from 0.975 to 0.968. However, more than 94% of the samples in each class were correctly labeled.

The demonstrated results will make it possible to analyze changes in chemical bonds by the Raman spectroscopy method during various processes, for example, conjugation, to study complex mixtures of substances, even with small additions of the components of interest.

## 4. Conclusions

Ensemble machine learning methods are advanced techniques that enable the integration of multiple individual estimators into a unified model. Two ensemble methods, namely, random forest (RF) and gradient boosting (GB), were used to detect small changes in the Raman spectrum after adding hyaluronic acid (HA) to whey protein isolate (WPI). Because the amount of HA added was small (0.1%, 0.25%, 0.5%), WPI’s normalized Raman band intensity was not directly affected. However, using the feature_importances_ property of the random forest or gradient boosting could explicitly identify the wavenumbers (features) of the data set based on the ensemble method that made the decision.

From the point of view of machine learning, this task can be represented as both regression and classification. The classification model showed greater accuracy (0.975 in the RF, 0.985 in the GB) compared with the regression model (R^2^ = 0.812 in the RF, R^2^ = 0.925 in the GB). The choice of the type of problem to be solved depends on the requirements. If subsequent measurements of other additive concentrations of samples are not planned, training classification models may be preferred. However, if machine learning results can be used in the form of calibration curves to determine the unknown concentration of additives using the Raman spectrum, the choice of regression models would be beneficial.

One way to improve the predictive model performance is to reduce the source data noise using principal component analysis (PCA). For example, when the number of components was set to 0.95 (by preserving 95% of the data’s explained variance), the number of features was reduced from 626 to 480 and the accuracy of the regression model was improved (R^2^ = 0.963 in the RF, R^2^ = 0.976 in the GB). The negative aspect of using PCA data converted to training models is that the new feature is a linear combination of the original feature. Therefore, it is impossible to directly identify the chemical bonds whose vibrations are recorded in Raman spectroscopy and which undergo the greatest changes after the addition of substances.

The addition of HA to WPI resulted in changes mainly around 1003 cm^−1^ (which corresponds to the ring breath of phenylalanine) and 1400 cm^−1^ (which corresponds to aspartic and glutamic acids and the C=O stretch of COO^−^), as demonstrated by both the regression and classification models based on the random forest and gradient boosting. For the regression models, changes in the Raman band at ∼1003 cm^−1^ accounted for more than 50% of the final prediction.

## Figures and Tables

**Table 1:** Raman bands of WPI with their assignments [34,43].

Wavenumber, cm^−1^	Assignment
760, 880, 1360	Tryptophan, indole ring
830, 855	Tyrosine, Fermi resonance between ring fundamental and overtone
1003	Phenylalanine, ring breath
1240	Amide III, N–H in-plane bend, C–N stretch
1400	Aspartic and glutamic acids, C=O stretch of COO^−^
1450, 1465	Aliphatic residues, C–H bending
1540	Amide II, N–H deformation
1667	Amide I, amide C=O stretch, N–H wag

## Data Availability

The datasets presented in this article are not readily available because the data are part of an ongoing study.

## Supplementary Materials

The following supporting information can be downloaded from https://www.mdpi.com/article/10.3390/polym16050666/s1—Figure S1: Raman maps of selected Raman bands (4×4 grid). Each row corresponds to a band at 1003, 1205, 1240, or 1401 cm^−1^. The normalized Raman intensity color bar is on the right. Each column corresponds to a sample of WPI, WPI + 0.1% HA, WPI + 0.25% HA, or WPI + 0.5% HA. The average of the 600 spectra normalized to the true amount of HA is shown in the bottom row. Gray lines correspond to selected Raman bands. Figure S2: The average of the 600 spectra normalized to the true amount of HA, with standard deviations calculated for each sample (**A**). Zoomed in 1200–1400 cm^−1^ range (**B**). The gray dashed and dotted lines correspond to wavenumbers with a feature importance calculated by RF and GB, respectively, as being greater than 1%. The matching lines are indicated as solid lines.

## Abbreviations

The following abbreviations are used in this manuscript:

WPI	whey protein isolate
HA	hyaluronic acid
RF	random forest
GB	gradient boosting
PCA	principal component analysis
FTIR	Fourier-transform infrared spectroscopy
