# Supplementary material for: Combination of Machine Learning and Raman Spectroscopy for Determination of the Complex of Whey Protein Isolate with Hyaluronic Acid
Source: Polymers (Basel). 2024 Feb 29;16(5):666. doi: 10.3390/polym16050666 (PMC10934111; doi:10.3390/polym16050666)
Supplement: Supplementary file 1 [file polymers-16-00666-s001.zip › polymers-2825209-supplementary.pdf]

Supplementary Materials: Combination of Machine Learning and Raman Spectroscopy for Determination of the Complex of Whey Protein Isolate with Hyaluronic Acid

Oksana A. Mayorova , Mariia S. Saveleva , Daniil N. Bratashov and Ekaterina S.Prikhozhdenko *

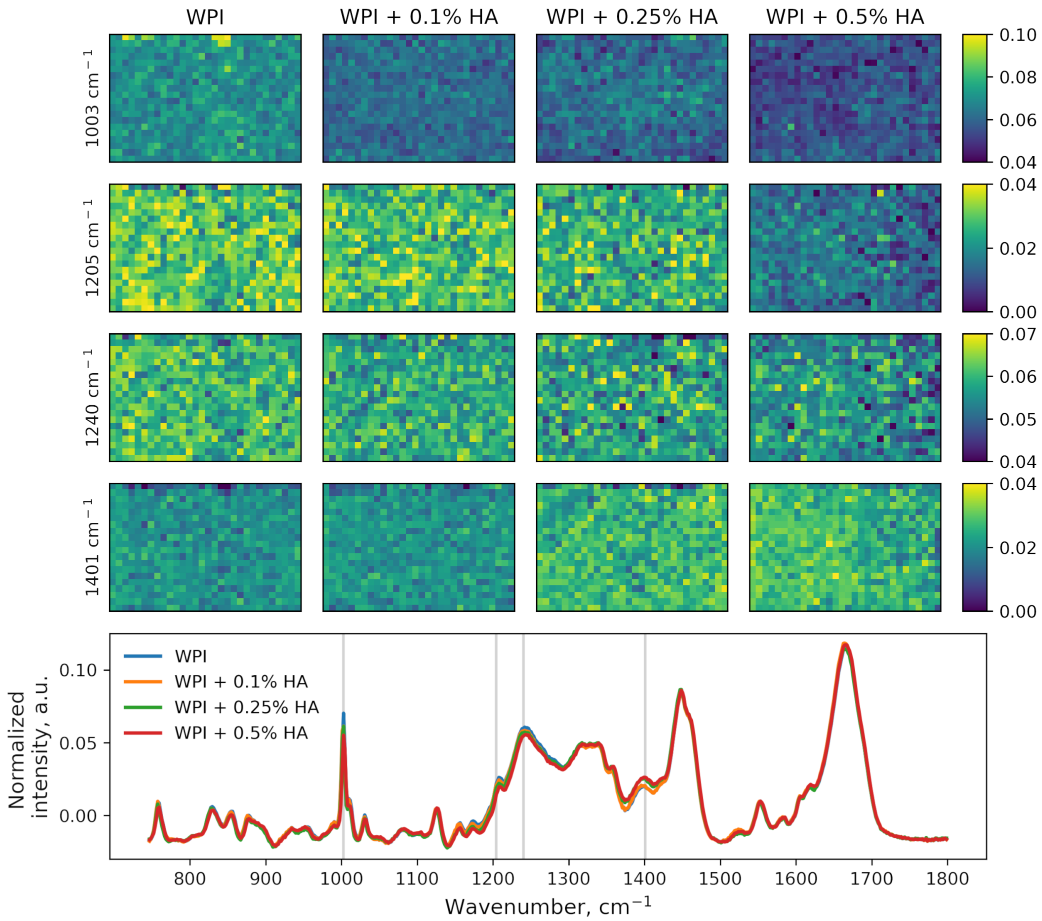

Figure S1. Raman maps of selected Raman bands (4 × 4 grid). Each row corresponds to a band at 1003, 1205, 1240 or 1401 cm^{-1}. The normalized Raman intensity color bar is on the right. Each column corresponds to a sample of WPI, WPI + 0.1% HA, WPI + 0.25% HA, or WPI + 0.5% HA. The average of the 600 normalized spectra to the true amount of HA is shown in the bottom row. Gray lines correspond to selected Raman bands.

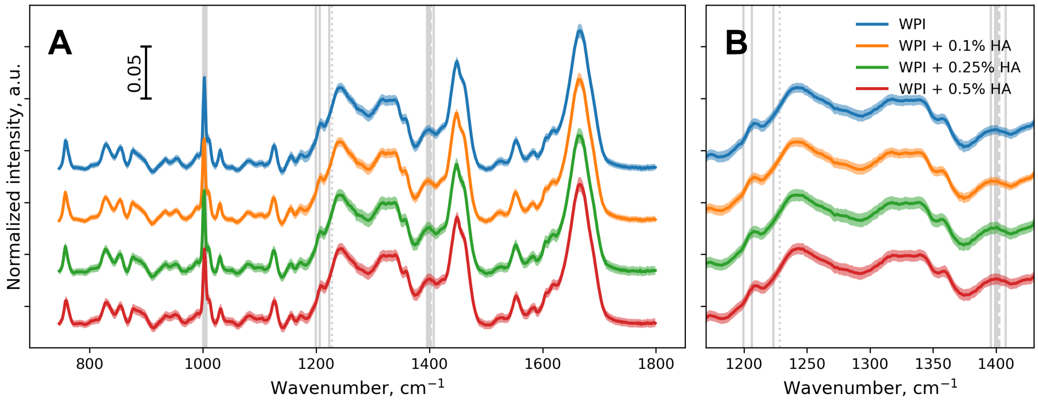

Figure S2. The average of the 600 normalized spectra to the true amount of HA with standard deviations calculated for each sample (**a**). Zoomed in 1200–1400 cm^{-1} range (**b**). The gray dashed and dotted lines correspond to wavenumbers with a feature importance calculated by RF and GB, respectively, greater than 1%. The matching lines are indicated as solid lines.
